# Role of Receptor-Interacting Protein 140 in human fat cells

**DOI:** 10.1186/1472-6823-10-1

**Published:** 2010-01-29

**Authors:** Niklas Mejhert, Jurga Laurencikiene, Amanda T Pettersson, Maria Kaaman, Britta M Stenson, Mikael Rydén, Ingrid Dahlman

**Affiliations:** 1 Department of Medicine, Huddinge, Lipid Laboratory, NVS, Karolinska Institutet, SE- 141 86 Stockholm, Sweden

## Abstract

**Background:**

Mice lacking *Receptor-interacting protein 140 (RIP140) *have reduced body fat which at least partly is mediated through increased lipid and glucose metabolism in adipose tissue. In humans, *RIP140 *is lower expressed in visceral white adipose tissue (WAT) of obese versus lean subjects. We investigated the role of *RIP140 *in human subcutaneous WAT, which is the major fat depot of the body.

**Methods:**

Messenger RNA levels of *RIP140 *were measured in samples of subcutaneous WAT from women with a wide variation in BMI and in different human WAT preparations. *RIP140 *mRNA was knocked down with siRNA in *in vitro *differentiated adipocytes and the impact on glucose transport and mRNA levels of target genes determined.

**Results:**

*RIP140 *mRNA levels in subcutaneous WAT were decreased among obese compared to lean women and increased by weight-loss, but did not associate with mitochondrial DNA copy number. *RIP140 *expression increased during adipocyte differentiation *in vitro *and was higher in isolated adipocytes compared to corresponding pieces of WAT. Knock down of *RIP140 *increased basal glucose transport and mRNA levels of *glucose transporter 4 *and *uncoupling protein-1*.

**Conclusions:**

Human *RIP140 *inhibits glucose uptake and the expression of genes promoting energy expenditure in the same fashion as the murine orthologue. Increased levels of human *RIP140 *in subcutaneous WAT of lean subjects may contribute to economize on energy stores. By contrast, the function and expression pattern does not support that *RIP140 *regulate human obesity.

## Background

Adipose tissue has a central role in regulating energy homeostasis. Maintenance of energy balance requires tightly regulated expression of gene networks that control metabolic functions in response to changing environmental conditions [1,2].

*Receptor-interacting protein 140 (RIP140) *is a nuclear receptor corepressor that in mice is expressed in several organs; however the mRNA levels in white adipose tissue (WAT) are higher than in other metabolically active tissues, such as brown adipose tissue (BAT), muscle, and liver [3-5]. The physiological function of *RIP140 *has been tested in *RIP140 *knock out (RIPKO) mice. These mice have a reduced body weight and body fat content, when compared to wild-type (WT) mice [3]. The lean phenotype of RIPKO mice is not explained by impaired adipogenesis, since *RIP140 *is not required for adipocyte differentiation [3]. Furthermore, RIPKO mice exhibit increased oxygen consumption, total fatty acid oxidation, glucose tolerance, insulin responsiveness upon high-fat feeding, and resistance to high-fat diet-induced obesity [3,5]. At the cellular level several genes, including *cell death-inducing DFFA-like effector a (CIDEA), uncoupling protein-1 (UCP-1)*, and *glucose transporter 4 (GLUT4) *are upregulated in adipocytes from RIPKO as compared to WT-mice. Thus in mice *RIP140 *seems to play an important role in energy homeostasis which at least in part can be explained by its action on glucose uptake as well as lipid metabolism in white fat cells.

Surprisingly few studies have examined the expression of *RIP140 *expression in human organs. We therefore searched the GEO profiles database http://www.ncbi.nlm.nih.gov/ for *RIP140 *mRNA expression in the human transcriptome. According to record GDS596 *RIP140 *mRNA is widely expressed in different human tissues with particularly high expression levels observed in lung, skeletal muscle, reproductive organs and brain. It has recently been reported that *RIP140 *mRNA and protein levels are decreased in visceral WAT of morbidly obese as compared to lean humans implying that human *RIP140 *may, just as its rodent orthologue, regulate adipose tissue metabolism [6]. However, the function and expression of *RIP140 *mRNA in human subcutaneous WAT, which comprises the main store of body fat, has to our knowledge not been reported.

Caution should be exercised when extrapolating data from mice to man when adipose tissue is compared. For example there are major species differences in the regulation of lipid metabolism in fat cells [7]. This study was conducted with the aim of elucidating if *RIP140 *might be involved in the regulation of the subcutaneous fat mass in humans and if *RIP140 *had similar function in human white fat cells as in murine adipocytes. To accomplish this we investigated if *RIP140 *was present in human white fat cells and related the expression of *RIP140 *in human subcutaneous WAT to adiposity. In order to mimic the effect of gene knock out in mice we silenced *RIP140 *expression in human *in vitro *differentiated adipocytes and analyzed the effects of decreased mRNA levels of *RIP140 *on glucose transport and a set of genes involved in the control of energy homeostasis.

## Methods

Subjects were recruited by local advertisement for the purpose of studying genes regulating obesity and fat cell function. Obesity was defined as having a BMI ≥ 30 kg/m^2^, whereas leanness was defined as having a BMI ≤ 25 kg/m^2^. Informed consent was received from all subjects involved in the study. The project was conducted in accordance with the guidelines in The Declaration of Helsinki and approved by the ethical committee at Karolinska University Hospital.

Paired samples of omental and abdominal subcutaneous WAT for mRNA measurements were available in cohort 1 comprising lean (N = 11; age 40 ± 14 years; BMI 24 ± 2 kg/m^2^) and obese (N = 22; age 43 ± 9 years; BMI 44 ± 4 kg/m^2^) women. The non-obese subjects were operated for uncomplicated gallstone disease and the obese with anti-obesity surgery as described previously [8]. These patients had been fasting overnight and only saline was given as an intravenous infusion until adipose tissue was removed [8].

Subcutaneous abdominal WAT biopsies for mRNA measurements were available from cohorts 2 and 3. WAT (0.5-2 g) were obtained in the morning after an overnight fast from needle biopsies under local anesthesia as described previously [9]. Cohort 2 (N = 10 women; age 39 ± 6 years; BMI 40 ± 6 kg/m^2^) were investigated before and after (1.5-4 years) weight reduction induced by intense anti-obesity therapy with anti-obesity surgery or behavioral modification. All subjects became non-obese and were reinvestigated when they were weight-stable. These subjects have been described before [10]. Cohort 3 comprised 34 women with a wide variation in BMI (N = 34; age 42 ± 12 years; BMI 30 ± 7 kg/m^2^) for which relative amounts of mitochondrial DNA (mtDNA) to nuclear DNA in subcutaneous WAT were determined by quantitative RT-PCR. To investigate if *RIP140 *levels differed between genders we investigated three men before (age 37 ± 11 years; BMI 38 ± 3 kg/m^2^) and after the anti-obesity treatment described above under cohort 2.

For the following adipocyte studies abdominal subcutaneous adipose tissue pieces were obtained as a waste product from plastic surgery. There was no selection on the basis of body fat content. WAT pieces were treated with collagenase as described elsewhere for isolation of the fat cells and the stroma vascular fraction, respectively [11]. Paired samples of isolated adipocytes and corresponding bits of subcutaneous WAT were available from five obese and five lean women (age 43 ± 14; BMI 28 ± 6 kg/m^2^). Cells harvested from the stroma vascular fraction were *in vitro *differentiated to adipocytes according to a method described previously [11]. *RIP140 *mRNA was quantified at day 4^th^, 8^th^, and 12^th ^of differentiation in primary adipocyte cultures from eight women (age 46 ± 13 years; BMI 27 ± 6 kg/m^2^). *in vitro *differentiated adipocytes were prepared from five women (age 44 ± 13 years; BMI 26 ± 2 kg/m^2^) for siRNA and glucose transport experiments. *RIP140 *protein was detected in subcutaneous WAT from five women (age 32 ± 4 years; BMI 24 ± 8 kg/m^2^).

WAT samples were brought to the laboratory in saline and either used immediately for *in vitro *studies or frozen in liquid nitrogen, and stored at -70°C until mRNA, DNA, and/or protein measurements were performed.

### Quantification of mitochondrial DNA copy number

The ratio of mtDNA to nuclear DNA reflects the tissue concentration of mtDNA per cell and was determined by quantitative RT-qPCR as described. Briefly, a 120 nucleotide-long mtDNA fragment within the mitochondrial *NADH dehydrogenase subunit 1 (ND1) *gene was used for quantification of mtDNA. The PCR fragment has previously been cloned into a plasmid. Plasmid standards of known copy number were used to generate a log-linear standard curve, from which the *ND1 *copy numbers of studied samples could be determined by RT-qPCR. A 120 bp region of the nuclear gene *lipoprotein lipase (LPL) *was used to normalize results. Plasmid standard curves containing the *LPL *fragment were used to determine cell number of studied samples [12,13].

### Protein expression

Approximately 300 mg of subcutaneous WAT was lysed in 600 μl protein lysis buffer (1% Triton-X 100, Tris-HCl pH 7.6 and 150 mmol/L NaCl, 4°C), supplemented with protease inhibitors (1 mmol/L phenylmethylsulfonyl fluoride and Complete^® ^(Boehringer, Mannheim, Germany)), and homogenized. The lysed tissue was centrifuged at 14 000 RPM for 30 minutes after which the infranatant was collected. Protein content was assayed spectrophotometrically using the BCA Protein Assay Reagent Kit (PIERCE, Rockford, IL) on 96-well microtiter plates with bovine serum albumin (BSA) (SIGMA, St Louis, MO) as standard. To test if proteins remained in the fat cake following protein extraction the fat cake was removed and subjected to methanol-chloroform (Merck, Darmstadt, Germany) extraction which effectively collects all proteins [14]. These extracted proteins were dissolved in 600 μl protein lysis buffer (same as above). Protein levels in fat cake extracts were below the detection limit. After optimization the following conditions were chosen. 100 mg of total cellular protein was loaded on polyacrylamide gels and separated by standard 10% sodium dodecyl sulphate-polyacrylamide gel electrophoresis. Preadipocytes were used as a negative control since *RIP140 *expression is induced in later stages of differentiation. Gels were transferred to polyvinylidine fluoride membranes (Amersham Biosciences, Little Chalfont, UK). For *RIP140 *detection the blot was blocked for 1 h at room temperature in Tris-buffered saline with 0.1% Tween-20 (TBS-T) and 3% BSA (SIGMA). This was followed by an overnight incubation at 4°C in the presence of 4 μg/ml antibody directed against *RIP140 *(R 5027, SIGMA). The following day the membrane was rinsed in TBS-T and secondary α-rabbit antibodies conjugated to horseradish peroxidase (SIGMA) were added. The membrane was incubated in room temperature for one hour and after that rinsed with TBS-T. Antigen-antibody complexes were detected by chemiluminescence using a kit from LumiGLO^® ^(Cell Signaling Technology, Danvers, MA) and specific bands were detected using a Chemidoc XRS system (Bio-Rad Laboratories Inc., Hercules, CA).

### Small interfering RNA mediated knock down

Cells harvested from the stroma vascular fraction obtained after collagenase treatment of subcutaneous WAT were *in vitro *differentiated to adipocytes according to a method described previously [11] and treated with siRNA as described [15]. Briefly, 200 000 cells/well were seeded in a 12-well plate and *in vitro *differentiated for twelve days at 37°C in 5% CO_2_. On the tenth day of differentiation cells were treated with 100 nM ON-TARGETplus SMARTpool *RIP140 *(L-006686-00) small interfering RNA (siRNA) (Thermo Fisher Scientific, Lafayette, CO) and 9 μl HiPerFect Transfection Reagent (Qiagen, Hilden, Germany) according to the manufacturer's protocols. These conditions were chosen after careful optimization. To control for unspecific effects of siRNA treatment, control cells were treated with AllStars Negative Control siRNA (Qiagen). After 48 hours cells were lysed for isolation of RNA using 350 μl lysis buffer containing RA1 lysis buffer (Macherey-Nagel, Duren, Germany) and 3.5 μl β-mercaptoethanol.

### Glucose uptake

*In vitro *differentiated human adipocytes were treated with *RIP140 *or non-silencing siRNA as described above. 48 hours post transfection cells were washed with glucose-free DMEM (Biochrom, Berlin, Germany) and incubated at 37°C for three hours in medium consisting of 1:1 glucose-free DMEM and HAM/F12 (Gibco, Invitrogen, Carlsbad, CA). To determine net insulin effect on glucose transport, insulin was added to cells at a final concentration of 10^-6 ^M followed by incubation at 37°C for 15 minutes. One μCi of 2-Deoxy-D- [1-3H] glucose (Amersham Biosciences) per 1 ml medium was added to the cells followed by 20 minutes incubation at 37°C. Cells were washed in ice cold PBS, lysed in 0.1% SDS-H_2_O, and radioactivity was measured in liquid scintillant in a β-counter 1214 Rackbeta LKB (Wallac, PerkinElmer Life Sciences, Waltham, MA) the following day.

### RNA preparation

Total RNA was extracted from isolated adipocytes (200 μl) and corresponding pieces of subcutaneous WAT (300 mg), as well as *in vitro *differentiated adipocytes (200 μl) using the NucleoSpin^® ^RNA II (Macherey-Nagel) kit. RNA concentration and purity was measured spectrophotometrically using a Nanodrop ND-1000 Spectrophotometer (Thermo Fisher Scientific). Half a microgram of total RNA was reverse transcribed using Omniscript First-strand cDNA synthesis kit (Qiagen) and random hexamer primers (Invitrogen). RNA purified from *in vitro *differentiated adipocytes were reversed transcribed using iScript™ cDNA synthesis kit (Bio-Rad Laboratories Inc.), with some modifications. Purified total RNA, RNase-free water, and 5× iScript reaction mixes were preheated for 15 minutes at 55°C. After preheating tubes were put on ice for one minute and iScript reverse transcriptase was added.

### Quantitative real time PCR

For TaqMan assays 10 ng of cDNA was mixed with 2× TaqMan Universal PCR Master Mix (Applied Biosystems, Foster City, CA) and TaqMan^@ ^Gene Expression Assays (Applied Biosystems) in a final volume of 20 μl. mRNA was analyzed for *RIP140 *(Hs00942766_s1), *glucose transporter 1 (GLUT1) *(Hs00197884_m1), *GLUT4 *(Hs00168966_m1), *CIDEA *(Hs00154455_m1), *UCP-1 *(Hs00222453_m1), *PPARγ co-activator 1a (PGC-1α) *(Hs00173304_m1), *low density lipoprotein receptor-related protein 10 (LRP10) *(Hs00204094_m1), and *18S *(Hs99999901_s1). For SYBR Green assays, 5 ng of cDNA was mixed with 2× iQ SYBR Green Supermix (Eurogentec S.A., Ougrée, Belgium) and primers (Invitrogen) in a final volume of 25 μl. Primer pairs were designed to span exon-intron boundaries and to be specific for the mRNA to be amplified according to BLAST searches. The following primers were used for specific mRNA quantification: *RIP140 *(forward primer 5'-TGA CCA AAA CCA ACC CAA TAC T-3', reverse primer 5'-GAC CTG TGA GAC ACT TTC AGC A-3'), *PPARγ *(forward primer 5'-ACA AGG CCA TTT TCT CAA ACG-3', reverse primer 5'-CGG AGA GAT CCA CGG AGC-3'), *hormone-sensitive lipase (HSL) *(forward primer 5'-GGA AGT GCT ATC GTC TCT GG-3', reverse primer 5'-GGC AGT CAG TGG CAT CTC-3'), *LRP10 *(forward primer 5'-GAT GGA GGC TGA GAT TGT G-3', reverse primer 5'-GAG TCA TAT CCT GGC GTA AG-3'), and *18S *(forward primer 5'-TGA CTC AAC ACG GGA AAC C-3', reverse primer 5'-TCG CTC CAC CAA CTA AGA AC-3'). Dissociation curve analyses and agarose gel electrophoresis were used to validate that one single amplicon was amplified. Quantitative real-time PCR was performed using an iCycler IQ™ (Bio-Rad Laboratories Inc.). PCR efficiency in all runs was close to 100% and samples were run in duplicate. Expression of mRNA was normalized to an internal reference gene, *18S *(isolated adipocytes and corresponding bits of subcutaneous WAT) or *LRP-10 *(*in vitro differentiated *adipocytes) by using a comparative Ct method, i.e. 2^ΔCt-target gene^/2^ΔCt-reference gene^. The patient with the highest Ct value for the target gene was used as a calibrator from which all other Ct values for the target gene and reference gene, respectively, were subtracted.

### Statistical analysis

The mRNA levels between two groups were compared with paired Student's t-test. When comparing three groups, results were evaluated with ANOVA. Correlation between *RIP140 *mRNA levels and mtDNA copy number was studied with multiple regression analysis with age and BMI as covariate. If necessary, *RIP140 *levels were log_10_-transformed to become normally distributed. Values were presented as mean ± SD. All statistics were calculated with Statview (version 5.01, SAS Institute, Cary, NC).

## Results

### Subcutaneous WAT *RIP140 *mRNA levels are inversely correlated to obesity

**Figure 1:**
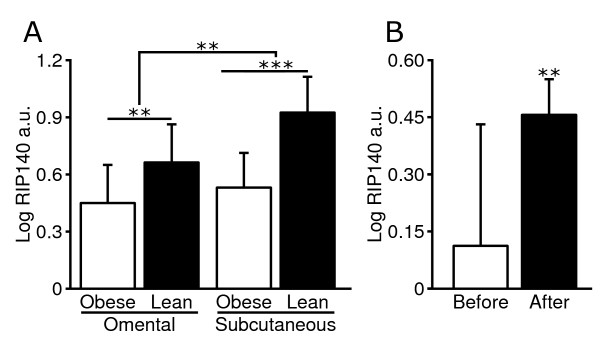
***RIP140 *mRNA expression in human WAT and obesity**. **A) ***RIP140 *mRNA levels were measured in omental and subcutaneous WAT from 22 obese and 11 lean women. **B) **Subcutaneous WAT *RIP140 *mRNA levels were investigated before and after weight-loss to a weight-stable non-obese state (subjects, N = 10). All samples were run in duplicate and normalized to the reference gene *18S*. Arbitrary units (a.u.) are presented as mean ± standard deviation and were log_10_-transformed to become normally distributed. **, P < 0.01 and ***, P < 0.001.

We first assessed abdominal subcutaneous WAT *RIP140 *mRNA levels in relationship to adiposity. *RIP140 *mRNA levels were decreased approximately 50% in obese compared to lean women in subcutaneous WAT (cohort 1, P < 0.001) (Figure 1A). We also confirmed decreased levels (25%) of *RIP140 *mRNA in visceral WAT of obese subjects (cohort 1, P < 0.01) (Figure 1A). Levels of *RIP140 *mRNA were lower in visceral as compared to subcutaneous WAT, and this was due to a difference in lean subjects only (Figure 1A). No difference between WAT depots were observed in obese subjects. Expression of *RIP140 *mRNA in subcutaneous WAT increased 2.5-fold after long term weight-loss to a weight-stable, lean state (cohort 2, P < 0.01) (Figure 1B). There was no difference in *RIP140 *mRNA expression between genders before (women 0.11 ± 0.34 vs. men 0.09 ± 0.13 log_10 _AU) or after (women 0.42 ± 0.10 vs. men 0.30 ± 0.12 log_10 _AU) weight-loss.

Considering that *RIP140 *has been implicated in regulation of energy expenditure and mitochondrial function in mice, we related subcutaneous WAT *RIP140 *mRNA levels to mtDNA copy number, which is a simple measure of mitochondrial mass that can be used to screen mitochondrial content in a large number of samples [5,13]. *RIP140 *mRNA levels in subcutaneous WAT did not associate with mtDNA copy number in 34 investigated women (results not shown).

### *RIP140 *mRNA and protein in WAT

**Figure 2:**
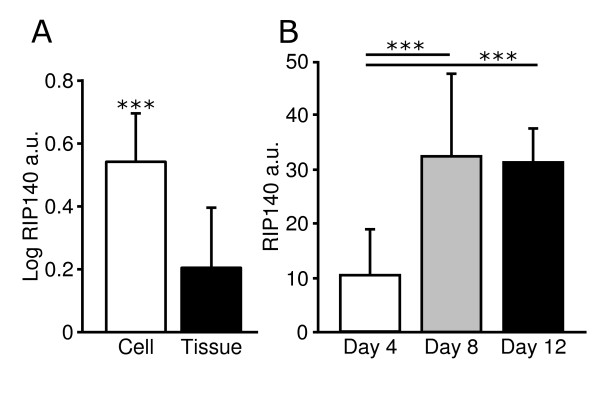
**Localization of human *RIP140 *in WAT**. **A) ***RIP140 *mRNA levels were measured in isolated adipocytes and corresponding bits of subcutaneous WAT from ten women. **B) ***RIP140 *mRNA expression was quantified at the 4^th^, 8^th^, and 12^th ^day of adipocyte differentiation *in vitro*. *RIP140 *arbitrary units (a.u.) were calculated as 2^ΔCt-target gene/^2^ΔCt-reference gene ^using *18S *(isolated adipocytes and corresponding WAT) and *LRP10 *(cultured adipocytes) as reference genes. Values are presented as mean ± SD and were log_10_-transformed to become normally distributed when needed. ***, P < 0.001.

**Figure 3:**
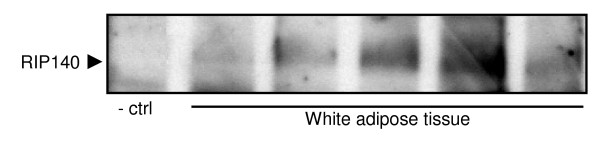
**Detection of *RIP140 *protein with Western blot in subcutaneous WAT of five women**. Preadipocytes were used as a negative control (-ctrl).

We next evaluated the distribution of *RIP140 *in subcutaneous WAT. *RIP140 *mRNA expression was increased almost twofold in isolated adipocytes compared to corresponding intact pieces of WAT (P < 0.001) (Figure 2A). Furthermore, obesity was associated with reduced levels of *RIP140 *mRNA in adipocytes (obese N = 5, 0.87 ± 0.12 log_10 _AU vs. lean N = 5, 1.12 ± 0.10 log_10 _AU, P > 0.01). During *in vitro *human adipocyte differentiation *RIP140 *levels were up-regulated during the 8^th ^and 12^th ^day compared to the 4^th ^day of differentiation (P < 0.001 for both). There was no significant change in expression when comparing the 8^th ^and 12^th ^day (Figure 2B). *RIP140 *protein was detected by western blot analysis in subcutaneous WAT with preadipocytes as a negative control (Figure 3).

### *RIP140 *knock down increases glucose uptake and expression of *UCP*-1 and *GLUT4*

**Figure 4:**
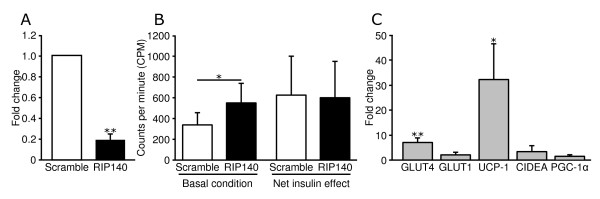
**Effect of *RIP140 *silencing on human in vitro differentiated adipocytes**. **A) ***RIP140 *mRNA levels in *RIP140 *siRNA and non-silencing siRNA (scramble) treated cells. **B) **Glucose transport, measured as counts per minute (CPM), was determined 48 hours post siRNA transfection. Net insulin effect was calculated by subtracting insulin-stimulated glucose uptake with basal glucose uptake. **C) **Specific mRNA levels were measured 48 h after siRNA mediated *RIP140 *knock down. In A and C, values are presented as fold change versus non-silencing siRNA (scramble). Data presented are mean ± SD of five independent experiments. *, P < 0.05 and **, P < 0.01.

To elucidate the biological role of *RIP140 *we silenced *RIP140 *in *in vitro *differentiated human adipocytes by siRNA. Depletion of *RIP140 *mRNA levels was confirmed by real-time PCR to be significantly knocked down by ~80% (p < 0.01) (Figure 4A). We then investigated how this knock down affected glucose transport and mRNA levels for a set of genes involved in glucose metabolism and energy expenditure. Basal glucose transport was significantly up regulated in *RIP140 *knock down adipocytes compared to cells transfected with non-silencing siRNA (p < 0.05), while the net insulin effect was unaffected (Figure 4B). In general, insulin-stimulated glucose uptake was significantly higher than basal glucose uptake in non-silencing as well as *RIP140 *siRNA transfected cells and individuals with high basal glucose uptake had high insulin stimulated glucose uptake.

*RIP140 *silencing significantly increased mRNA levels of *GLUT4 *and *UCP-1 *compared to control cells (P < 0.01 for both) (Figure 4C). Fold change vs. non-silencing control siRNA was for *GLUT4 *7.2 ± 1.7 and for *UCP-1 *32.2 ± 14.5. A trend toward increased mRNA levels for *GLUT1 *and *CIDEA *was observed (P = 0.0594 and P = 0.052, respectively). Fold change vs. non-silencing control siRNA was for *GLUT1 *2.1 ± 1.0 and for *CIDEA *3.5 ± 2.2. In contrast, *PGC-1α, PPARγ*, and *HSL *mRNA levels remained unchanged (Figure 4C).

## Discussion

To the best of our knowledge this is the first study investigating the function of *RIP140 *in human subcutaneous WAT, which is the major fat depot of the body. In mice it appears that the *RIP140 *is involved in glucose metabolism and energy expenditure of WAT [3-5,16,17]. We have shown that *RIP140 *mRNA levels in subcutaneous WAT, as has previously been reported for visceral WAT [6], are inversely correlated with measures of adiposity and are enriched in fat cells of adipose tissue. Moreover, *RIP140 *reduce basal glucose transport and expression of the genes *GLUT4 *and *UCP*-1.

*RIP140 *is enriched in isolated fat cells than intact WAT. This suggests that its major function is confined to the adipocytes of WAT although a role in stroma vascular cells is not to be excluded. Furthermore, human *RIP140 *mRNA expression increased during adipocyte differentiation, which is in agreement with results in mice [3-5,18]. Since mouse *RIP140 *has no impact on the differentiation process, we focused on the importance of *RIP140 *for metabolism in the differentiated human white fat cells. Silencing of *RIP140 *increased basal glucose transport, but had no impact on the effect of insulin on glucose uptake. In 3T3-L1 adipocytes, inhibition of *RIP140 *by siRNA has been reported to increase insulin-stimulated glucose uptake [5]. However, at an early stage of adipocyte differentiation, basal glucose uptake was also increased [5]. Furthermore, we observed that primarily mRNA levels of the glucose transporter *GLUT4 *but not *GLUT1 *were increased by *RIP140 *silencing in human fat cells, which is in line with data from knock out mice [5]. An impact on *GLUT1 *expression could not be excluded but was marginal compared to the impact on *GLUT4 *expression. Thus, it appears that *RIP140 *in both human and mice acts as an inhibitor of glucose uptake although there may be some differences in the action of insulin on glucose uptake between the different in vitro cell systems. The mechanisms leading to increased basal glucose transport in *RIP140*-depleted human adipocytes are unknown. Potential mechanisms involve post-translational effects on *GLUT1 *protein or involvement of other *GLUT*-proteins. Additional experiments are needed to elucidate how *RIP140 *regulates basal glucose uptake. Similarly, additional experiments are necessary to clarify whether *GLUT4 *is directly regulated by *RIP140 *and what other transcriptional regulators are involved, or if altered *GLUT4 *expression is mediated through changes in adipocyte metabolism. In 3T3-L1 adipocytes, the effects of *RIP140 *on glucose uptake are at least partly mediated via the transcription factor *Estrogen-related receptor alpha (ERRα) *[5]. *ERRα *is an important regulator of genes in energy metabolism [5]. Loss of *ERRα *prevents increased *GLUT4 *expression and deoxyglucose uptake in *RIP140 *depleted adipocytes. Furthermore, *ERRα *expression is increased in *RIP140*-depleted adipocytes [5].

*RIP140 *silencing in human *in vitro *differentiated adipocytes led to increased mRNA levels of *UCP-1*, but not *PGC-1α*, *PPARγ*, and *HSL*. These results confirm previous data from knock out mice [3,4]. The up-regulation of *CIDEA *expression was non-significant in our study (P = 0.0525). However, it is quite conceivable that *RIP140 *regulates *CIDEA *expression in human WAT since our results are in line with previous observations in mice [3-5]. In a one sided analysis, which is justified for confirmatory data, *CIDEA *mRNA levels were induced significantly by *RIP140 *silencing (P < 0.05). Altogether, *RIP140 *had a smaller impact on *CIDEA *mRNA levels in humans as compared to mice. One possible explanation for this discrepancy is the difference in *CIDEA *expression between mice and human. Human *CIDEA *is expressed in WAT in contrast to mice where *CIDEA *is widely expressed in BAT but most often not detected in WAT [19-21]. *UCP-1 *encodes a member of the mitochondrial proton carrier family of proteins and is expressed in BAT, but only to a limited extent in WAT [22]. Elevated *UCP-1 *levels are thought to convert WAT towards a browner phenotype by allowing mitochondrial respiration to occur in the absence of ATP-synthesis with dissipation of the produced energy as heat. *CIDEA *has also been implicated as a regulator of energy expenditure in both mice and humans [21,23]. Thus, our results support the notion that inhibition of human *RIP140*, similar to the murine homologue, increases the expression of genes involved in the regulation of energy expenditure. In our study we found no association between *RIP140 *mRNA levels and mtDNA copy number, which is in agreement with a study performed in mice [4]. This together with the elevated levels of *UCP-1 *suggests that *RIP140 *influences the function of existing mitochondria rather than biogenesis.

As has previously been reported in visceral WAT [6], abdominal subcutaneous *RIP140 *mRNA levels were reduced in obesity. Considering the functions ascribed to lack of *RIP140 *in *in vitro *studies, i.e. increased glucose uptake and elevated energy expenditure, it is difficult to link reduced *RIP140 *levels in obesity with a primary role in adiposity. We therefore consider the alternative that the physiological role of increased levels of *RIP140 *in lean subjects functions to economize on energy stores more likely. On the other hand, down regulation of *RIP140 *in obesity could be accompanied by changes in expression of other transcription factors and co-factors, which could lead to a different metabolic outcome than down regulation of *RIP140 *in cell cultures.

## Conclusion

In conclusion, in agreement with its mouse homologue human *RIP140 *is involved in the control of energy homeostasis of white fat cells by inhibiting glucose uptake and genes regulating energy expenditure. Furthermore, increased *RIP140 *expression in lean and after weight reduction may be a mechanism to economize on energy stores. By contrast, the function and expression pattern does not support that *RIP140 *regulate human obesity.

## Competing interests

The authors declare that they have no competing interests.

## Authors' contributions

All authors have read and approved the manuscript. NM and ID have made substantial contributions to the study design, acquisition and analysis of data. JL, BMS and MR participated in the optimization of the western blot and the cDNA-synthesis as well as revised the manuscript. ATP and MK participated in the design of the siRNA and mtDNA copy number experiments and helped to draft the manuscript.

## Pre-publication history

The pre-publication history for this paper can be accessed here:

http://www.biomedcentral.com/1472-6823/10/1/prepub
